# Association between SNPs in microRNA machinery genes and gastric cancer susceptibility, invasion, and metastasis in Chinese Han population

**DOI:** 10.18632/oncotarget.21199

**Published:** 2017-09-23

**Authors:** Xingbo Song, Huiyu Zhong, Qian Wu, Minjin Wang, Juan Zhou, Yi Zhou, Xiaojun Lu, Binwu Ying

**Affiliations:** ^1^ Department of Laboratory Medicine, West China Hospital, Sichuan University, Chengdu, Sichuan, 610041, China

**Keywords:** microRNA, single nucleotide polymorphism, gastric cancer, tumor invasion and metastasis

## Abstract

**Objective:**

The present study investigates the influence of genetic variants in miRNA machinery genes (*DROSHA, DICER, AGO1*, and *GEMIN4*) on gastric cancer in Chinese Han population, further revealing the genetic mechanisms of gastric cancer occurrence and development.

**Methods:**

Genotyping of single nucleotide polymorphisms (SNPs) was performed in 628 patients with GC and 502 frequency-matched (age and gender) controls by the high resolution melting (HRM) method.

**Results:**

The SNPs rs3742330 (*DICER*) and rs7813 (*GEMIN4*) were associated with susceptibility to gastric cancer (*P* = 0.002 and 0.010, respectively). Stratified analysis showed that the G allele of rs3742330 and genotype TT as well as T allele of rs7813 were associated with a later stage of gastric cancer (*P*=0.027, 0.032 and 0.018, respectively). Furthermore, the genotype TT and T allele of rs7813 appeared to be associated with a higher level of lymphatic metastasis of gastric cancer (*P*=0.021 and 0.030, respectively), while the genotype AA and A allele of rs636832 (*AGO1*) were correlated with a lower level of lymphatic metastasis of gastric cancer (*P*=0.016 and 0.041, respectively). There was no significant association between rs10719 (*DROSHA*) and gastric cancer.

**Conclusion:**

The present data demonstrated that genetic variants in miRNA machinery genes had a significant association with GC susceptibility (*DICER* and *GEMIN4*) and malignant behavior such as tumor stage (*DICER* and *GEMIN4*) and lymphatic metastasis of GC (*GEMIN4* and *AGO1*) in Chinese Han population.

## INTRODUCTION

Gastric cancer (GC) is the most common malignant tumor of the digestive system and the third leading cause of cancer mortality worldwide [1]. The development and progression of GC are affected by the interaction between environmental factors and individual genetic factors. Factors including *Helicobacter pylori* infection, salted food, drinking, smoking and so on were proved by classic epidemiological studies to be the main risk factors of GC [2], but there are differences in GC susceptibility and tumor progression between individuals. These differences are associated with gene polymorphisms.

MicroRNAs (miRNAs) are small single-stranded RNA molecules of about 21-23 nucleotides (nt) in length. Recently, miRNA is widely recognized as regulators of gene expression and regulate about 30% genes in humans [3–6]. The process of miRNA synthesis begins within the nucleus where RNA polymerase II converts miRNA into pri-miRNA. The pri-miRNA is then processed into a precursor of ∼70 nt in length with a hairpin structure by a DNA endonuclease enzyme named DROSHA (RNase III) as well as its cofactor DGCR8; this precursor is called pre-miRNA. At the same time, DROSHA and DGCR8 protein constitute a microprocessor complex in the formation of pre-miRNA. Next, Exportin-5/Ran-GTP complex transfers pre-miRNA to the cytoplasm, and pre-miRNA is then cut into miRNA duplexes (about 20 bp) by the TAR RNA binding protein (TRBP)-related DICER [7–11]. One strand of the miRNA duplexes integrates into miRNA-induced silencing complex (miRISC) and becomes mature miRNA. The miRISC contains proteins including AGO1-4, GEMIN3, and GEMIN4 that participate in mRNA inhibition or shearing of target mRNA [12–15]. Therefore, genetic polymorphisms in microRNA machinery genes could lead to abnormal expression of miRNAs and in turn affect the expression level of target genes, thus becoming the risk factor of disease such as tumor. Currently, there is little research exploring the influence of single nucleotide polymorphisms (SNPs) in miRNA machinery genes on GC susceptibility, invasion, and metastasis.

This research chose SNP loci in classical miRNA machinery genes (rs3742330 in *DICER*, rs3744741 and rs7813 in *GEMIN4*, rs10719 in *DROSHA*, and rs636832 in *AGO1*) by using a candidate gene-based approach to genetically explore the effect of variants in miRNA machinery genes on GC susceptibility, invasion, and metastasis in Chinese Han population. In addition, the findings of this study might provide the basis for further revealing the specific mechanisms by which genetic variants of these genes participate in the occurrence and development of GC. Additional *in-silico* studies were used to assess the possible functional significance and miRNA-binding of the positive polymorphisms.

## RESULTS

### Demographic characteristics of the study participants

The demographic characteristics of 628 cases and 502 controls are presented in Table 1. The average age and sex had no significant differences between the patient group and control group (*P*=0.727, 0.577 respectively) and for smoking status and drinking status (*P*=0.297, 0.631 respectively). All the participants were from Chinese population.

**Table 1:** Basic demographic data of subjects and clinical characteristics of GC cases

Parameters	Case		Control		*P*
	n	Frequencies (%)	n	Frequencies (%)	
Age (year, mean ± SD)	56.5±12.1		56.2±12.2		0.727
Gender					
Male	418	66.6	323	64.3	0.577
Female	210	33.4	179	35.7	
Smoking Status					
Non-smokers	365	58.1	297	59.2	0.297
Former Smokers	139	22.1	125	24.9	
Current Smokers	124	19.7	80	15.9	
Drinking status					
Non-drinker	437	69.6	348	69.3	0.631
Light Drinkers	93	14.8	66	13.2	
Heavy Drinkers	98	15.6	88	17.5	
Tumor size (diameter)					
<5 cm	207	33.0			
5-10 cm	204	32.5			
≥10 cm	56	8.9			
N.A.	161	25.6			
Tumor stages					
1a	64	10.2			
1b	36	5.7			
2a	41	6.5			
2b	72	11.5			
3a	55	8.8			
3b	71	11.3			
3c	109	17.4			
4	180	28.7			
Degree of differentiation					
Low	433	68.9			
Medium	185	29.5			
High	10	1.6			
Lymphatic metastasis					
0	151	24.0			
1	103	16.4			
2	109	17.4			
3a	124	19.7			
3b	61	9.7			
N.A.	80	12.7			

N.A. data not available.

### The relationship between SNPs in miRNA machinery genes and GC susceptibility

Genotyping of five SNPs was successfully completed for the cases and controls. Genotype distribution of rs3744741 in patient group was not in accordance with the Hardy-Weinberg equilibrium (HWE) (χ2=10.18, *P*=0.001), while the other 4 SNPs in either patient or control groups met HWE (*P* > 0.05 for all loci). Thus, the SNP rs3744741 was excluded from further analysis (data not shown). Table 2 shows the genotype distributions and allele frequencies of the 4 SNPs in miRNA machinery genes between cases and controls.

**Table 2:** Comparisons of gene polymorphisms between the case and control groups

SNP	Cases		Controls		OR (95% C.I.)*	P*
	N	%	N	%		
rs 3742330						
Genotype						
AA	273	43.5	177	35.3	1.00 (Reference)	
AG	284	45.2	246	49.0	0.75 (0.58-0.97)	**0.026**
GG	71	11.3	79	15.7	0.58 (0.39-0.86)	**0.004**
Allele						
A	830	66.1	600	59.8	1.00 (Reference)	
G	426	33.9	404	40.2	0.76 (0.64-0.91)	**0.002**
rs7813						
Genotype						
TT	261	41.6	241	48.0	1.00 (Reference)	
CT	294	46.8	222	44.2	1.22 (0.95-1.58)	0.110
CC	73	11.6	39	7.8	1.73 (1.11-2.71)	**0.011**
Allele						
T	816	65.0	704	70.1	1.00 (Reference)	
C	440	35.0	300	29.9	1.27 (1.05-1.52)	**0.010**
rs10719						
Genotype						
TT	314	50.0	248	49.4	1.00 (Reference)	
CT	257	40.9	205	40.8	0.99 (0.77-1.28)	0.938
CC	57	9.1	49	9.8	0.92 (0.59-1.42)	0.690
Allele						
T	885	70.5	701	69.8	1.00 (Reference)	
C	371	29.5	303	30.2	0.97 (0.81-1.17)	0.741
rs 636832						
Genotype						
AA	321	51.1	268	53.4	1.00 (Reference)	
AG	261	41.6	198	39.4	1.10 (0.85-1.42)	0.445
GG	46	7.3	36	7.2	1.07 (0.65-1.74)	0.785
Allele						
A	903	71.9	734	73.1	1.00 (Reference)	
G	353	28.1	270	26.9	1.06 (0.88-1.29)	0.521

* Adjusted by sex, age, smoking status, and drinking status.

As shown in Table 2, the minor allele (G allele) frequency of rs3742330 was 33.9% in cases and 40.2% in controls and was significantly different (OR= 0.76, 95% CI= 0.64-0.91), the *p*-value was 0.002; this indicated that the G allele could be a protective element for GC susceptibility. As expected, genotype GG and AG of rs3742330 had a significantly decreased risk of GC compared with AA genotype (*P*=0.004, OR= 0.58, 95% CI= 0.39-0.86 for GG versus AA, and *P*=0.026, OR= 0.75, 95% CI= 0.58-0.97 for AG versus AA).

Conversely, subjects carrying a CC genotype in rs7813 showed a significant increase in risk for GC than those carrying the TT genotype (*P*=0.011, OR = 1.73, 95% CI = 1.11-2.71), and it was suggested that the C allele of rs7813 may be associated with a higher risk of GC than T allele (*P*=0.010, OR=1.27, 95%CI=1.05-1.52). However, no significant differences in genotype distributions or allelic frequencies of rs10719 and rs636832 were demonstrated between the cases and controls. All the above data were adjusted by sex, age, smoking status, and drinking status.

### Stratified analysis for the SNP genotypes and clinicopathologic characteristics of GC patients

To demonstrate the association between SNP genotypes and clinicopathologic characteristics of GC, the cases were stratified into subgroups according to tumor size, tumor stages, degree of differentiation, and lymphatic metastasis. The results for each SNP are summarized in Tables 3-1, 3-2, 3-3 and 3-4, respectively.

**Table 3-1:** Stratified analysis for the association between rs3742330 and GC clinical characteristics

Clinical characteristics	Genotype			OR (95% C.I.)^*^	*P*	Allele		OR (95% C.I.)	*P*
	AA	AG	GG			A	G		
Tumor size									
<5 cm	100	86	21	1.00 (Reference)		286	128	1.00 (Reference)	
5-10 cm	83	94	27	0.73 (0.49-1.11)	0.120	260	148	0.79 (0.58-1.06)	0.104
≥10 cm	26	26	4	0.93 (0.49-1.75)	0.803	78	34	1.03 (0.64-1.66)	0.909
Tumor stages									
1a	29	30	5	1.00 (Reference)		88	40	1.00 (Reference)	
1b	14	18	4	0.77 (0.31-1.91)	0.533	46	26	0.80 (0.42-1.55)	0.483
2a	13	18	10	0.56 (0.23-1.37)	0.165	44	38	0.53 (0.28-0.97)	**0.027**
2b	38	29	5	1.35 (0.65-2.81)	0.385	74	39	0.86 (0.49-1.53)	0.590
3a	25	24	6	1.01 (0.46-2.21)	0.988	74	36	0.93 (0.52-1.67)	0.807
3b	29	32	10	0.83 (0.40-1.75)	0.601	90	52	0.79 (0.46-1.35)	0.353
3c	46	51	12	0.88 (0.45-1.72)	0.690	143	75	0.87 (0.53-1.42)	0.548
4	79	80	21	0.94 (0.51-1.74)	0.844	238	122	0.89 (0.56-1.40)	0.586
Degree of differentiation									
Low	187	198	47	1.00 (Reference)		572	292	1.00 (Reference)	
Medium	82	82	21	1.04 (0.73-1.50)	0.812	246	124	1.01 (0.78-1.32)	0.923
High	4	4	2			12	8		
Lymphatic metastasis									
0	66	72	13	1.00 (Reference)		204	98	1.00 (Reference)	
1	42	42	19	0.89 (0.52-1.52)	0.643	126	80	0.76 (0.51-1.11)	0.139
2	49	51	9	1.05 (0.62-1.78)	0.842	149	69	1.04 (0.70-1.53)	0.847
3a	62	50	12	1.29 (0.78-2.13)	0.298	174	74	1.13 (0.77-1.65)	0.511
3b	19	33	9	0.58 (0.30-1.14)	0.091	71	51	0.67 (0.42-1.06)	0.068

**Table 3-2:** Stratified analysis for the association between rs7813 and GC clinical characteristics

Clinical characteristics	Genotype			OR (95% C.I.)^*^	*P*	Allele		OR (95% C.I.)	*P*
	TT	CT	CC			T	C		
Tumor size									
<5 cm	81	105	21	1.00 (Reference)		267	147	1.00 (Reference)	
5-10 cm	88	94	22	1.18 (0.78-1.78)	0.409	270	138	1.08 (0.80-1.45)	0.612
≥10 cm	26	26	4	1.35 (0.71-2.55)	0.324	78	34	1.26 (0.79-2.03)	0.309
Tumor stages									
1a	20	37	7	1.00 (Reference)		77	51	1.00 (Reference)	
1b	15	13	8	1.57 (0.62-4.00)	0.295	43	29	0.98 (0.52-1.85)	0.952
2a	13	24	4	1.02 (0.40-2.58)	0.961	50	32	1.03 (0.56-1.90)	0.906
2b	33	30	9	1.86 (0.87-4.00)	0.082	96	48	1.32 (0.78-2.24)	0.265
3a	20	28	7	1.26 (0.55-2.89)	0.556	68	42	1.07 (0.61-1.87)	0.793
3b	24	38	9	1.12 (0.51-2.46)	0.601	86	56	1.02 (0.61-1.71)	0.946
3c	54	50	5	2.16 (1.08-4.36)	**0.019**	158	60	1.74 (1.07-2.84)	**0.018**
4	84	74	22	1.92 (1.01-3.69)	**0.032**	242	118	1.36 (0.88-2.10)	0.149
Degree of differentiation									
Low	178	203	52	1.00 (Reference)		559	307	1.00 (Reference)	
Medium	79	88	18	1.07 (0.74-1.54)	0.713	246	124	1.09 (0.84-1.42)	0.513
High	4	3	3			11	9		
Lymphatic metastasis									
0	50	80	21	1.00 (Reference)		180	122	1.00 (Reference)	
1	48	45	10	1.76 (1.02-3.05)	**0.030**	141	65	1.47 (1.00-2.17)	**0.042**
2	43	52	14	1.36 (0.76-2.27)	0.293	138	80	1.17 (0.80-1.70)	0.393
3a	58	54	12	1.78 (1.06-2.98)	**0.021**	170	78	1.48 (1.02-2.14)	**0.030**
3b	28	28	5	1.71 (0.89-3.29)	0.080	84	38	1.50 (0.94-2.40)	0.075

**Table 3-3:** Stratified analysis for the association between rs10719 and GC clinical characteristics

Clinical characteristics	Genotype			OR (95% C.I.)^*^	*P*	Allele		OR (95% C.I.)	*P*
	TT	CT	CC			T	C		
Tumor size									
<5 cm	106	83	18	1.00 (Reference)		295	119	1.00 (Reference)	
5-10 cm	104	79	21	0.99 (0.66-1.49)	0.963	287	121	0.96 (0.70-1.31)	0.773
≥10 cm	30	21	5	1.10 (0.58-2.07)	0.753	81	31	1.05 (0.65-1.73)	0.825
Tumor stages									
1a	26	32	6	1.00 (Reference)		84	44	1.00 (Reference)	
1b	20	14	2	1.83 (0.74-4.54)	0.150	54	18	1.57 (0.79-3.16)	0.169
2a	23	14	4	1.87 (0.78-4.47)	0.121	60	22	1.43 (0.74-2.75)	0.251
2b	34	33	5	1.31 (0.63-2.74)	0.439	101	43	1.23 (0.72-2.12)	0.426
3a	30	19	6	1.75 (0.79-3.88)	0.129	79	31	1.33 (0.74-2.41)	0.305
3b	37	24	10	1.59 (0.76-3.34)	0.182	98	44	1.17 (0.68-2.00)	0.553
3c	59	41	9	1.72 (0.88-3.38)	0.086	159	59	1.41 (0.86-2.32)	0.151
4	85	80	15	1.31 (0.73-2.43)	0.363	250	110	1.19 (0.76-1.87)	0.425
Degree of differentiation									
Low	215	179	39	1.00 (Reference)		609	257	1.00 (Reference)	
Medium	94	74	17	1.05 (0.73-1.50)	0.792	262	108	1.02 (0.73-1.35)	0.863
High	5	4	1			14	6		
Lymphatic metastasis									
0	69	69	13	1.00 (Reference)		207	95	1.00 (Reference)	
1	55	39	9	1.36 (0.80-2.32)	0.228	149	57	1.20 (0.80-1.81)	0.360
2	60	39	10	1.46 (0.86-2.46)	0.137	159	59	1.24 (0.83-1.85)	0.279
3a	67	46	11	1.40 (0.80-2.42)	0.169	180	68	1.21 (0.83-1.79)	0.302
3b	26	26	9	0.88 (0.46-1.68)	0.684	78	44	0.81 (0.51-1.44)	0.360

**Table 3-4:** Stratified analysis for the association between rs636832 and GC clinical characteristics

Clinical characteristics	Genotype			OR (95% C.I.)^*^	*P*	Allele		OR (95% C.I.)	*P*
	AA	AG	GG			A	G		
Tumor size									
<5 cm	96	94	17	1.00 (Reference)		286	128	1.00 (Reference)	
5-10 cm	106	86	12	1.25 (0.83-1.88)	0.258	298	110	1.21 (0.89-1.66)	0.211
≥10 cm	27	24	5	1.08 (0.57-2.03)	0.807	78	34	1.03 (0.64-1.66)	0.909
Tumor stages									
1a	35	26	3	1.00 (Reference)		96	32	1.00 (Reference)	
1b	23	12	1	1.47 (0.58-3.70)	0.371	58	14	1.38 (0.65-2.98)	0.370
2a	25	12	4	1.29 (0.54-3.11)	0.525	62	20	1.03 (0.52-2.07)	0.920
2b	36	30	6	1.83 (0.40-1.72)	0.585	102	42	0.81 (0.46-1.43)	0.441
3a	34	20	1	1.34 (0.60-2.99)	0.432	88	22	1.33 (0.69-2.58)	0.358
3b	31	36	4	0.64 (0.31-1.34)	0.201	98	44	0.74 (0.42-1.31)	0.275
3c	46	51	12	0.60 (0.31-1.18)	0.112	143	75	0.64 (0.38-1.06)	0.068
4	91	74	15	0.85 (0.46-1.56)	0.570	256	104	0.82 (0.50-1.33)	0.399
Degree of differentiation									
Low	211	191	31	1.00 (Reference)		613	253	1.00 (Reference)	
Medium	103	67	15	1.32 (0.92-1.90)	0.114	273	97	1.16 (0.88-1.54)	0.284
High	7	3	0			17	3		
Lymphatic metastasis									
0	88	56	7	1.00 (Reference)		232	70	1.00 (Reference)	
1	59	36	8	0.96 (0.56-1.65)	0.875	154	52	0.89 (0.58-1.38)	0.593
2	47	56	6	0.54 (0.32-0.92)	**0.016**	150	68	0.67 (0.44-1.00)	**0.041**
3a	61	47	16	0.69 (0.42-1.15)	0.132	169	79	0.65 (0.43-0.96)	**0.023**
3b	27	31	3	0.57 (0.30-1.08)	0.064	85	37	0.69 (0.42-1.14)	0.125

As shown in Table 3-1, the A allele of rs3742330 may decrease the risk of GC in stage 1b rather than 1a (*P*=0.027, OR=0.53, 95%CI=0.28-0.97). However, there was no significant difference found in the frequency of AA genotype. According to Table 3-2, individuals carrying TT genotype and T allele of rs7813 had an increased risk of GC in tumor stage 3c than stage 1a (*P*=0.019, OR=2.16, 95%CI=1.08-4.36; *P*=0.018, OR=1.74, 95%CI=1.07-2.84, respectively). In terms of the data, the TT genotype of rs7813 also increased the risk of GC in stage 4 than stage 1a (*P*=0.032, OR=1.92, 95%CI=1.01-3.69). For GC invasion and metastasis, the data in Table 3-2 indicated that the TT genotype and the T allele of rs7813 had a higher risk of lymphatic metastasis stage 1 or 3a than stage 0 (*P*=0.030, OR=1.76, 95%CI=1.02-3.05; *P*=0.042, OR=1.47, 95%CI=1.00-2.17 and *P*=0.021, OR=1.78, 95%CI=1.06-2.98; *P*=0.030, OR=1.48, 95%CI=1.02-2.14). With regard to rs636832, as shown in Table 3-4, it is suggested that the AA genotype and A allele had an association with a lower risk of lymphatic metastasis stage 2 compared with stage 0 (*P*=0.016, OR=0.54, 95%CI=0.32-0.92 and *P*=0.041, OR=0.67, 95%CI=0.44-1.00 respectively), similar to the A allele which had a lower risk of lymphatic metastasis stage 3a (*P*=0.023, OR=0.65, 95%CI=0.43-0.96). Stratified analysis of rs10719 showed no significant differences in tumor size, tumor stages, degree of differentiation, or lymphatic metastasis of GC (Table 3-3). To demonstrate the mechanisms of these associations, further study is urgently needed.

### *In-silico* analysis of microRNA-binding and function prediction

As for rs3742330, computational modeling suggested that this polymorphism was located in the potential target sequence of hsa-miR-632 in *DICER* 3'UTR region (Supplementary Figure 1). The G allele could reduce the affinity of microRNA-mRNA binding by disrupting the local structure of DICER mRNA, possibly leading an increased DICER expression. In addition rs7813(C>T, R1033C) was a missense variant in exon region of *GEMIN4*, which could alter the structure of GEMIN4 protein by turning Arginine into Cysteine (Supplementary Figure 2), thus reducing GEMIN4 expression. There was no function results for rs636832 obtained from the software.

## DISCUSSION

Individual genetic factors play an important role in susceptibility and progression of GC. miRNA is a small single-stranded RNA of 21-23 nt in length and is widely recognized as regulators of gene expression. miRNAs participate in a variety of important biological processes including cell cycle, cell differentiation, and cell proliferation and apoptosis [16]. Previous studies have confirmed that miRNAs play an important role in a wide variety of tumor biological behaviors such as tumor cell proliferation and apoptosis. Clinically, there is abnormal expression of different levels of miRNAs in cancer patients, indicating that miRNA has a large influence on the development of tumor [17–19]. Ahn DH [20] chose four SNPs in miRNA and analyzed the genotypes and allele frequencies of these SNPs in 461 Korean GC patients. The study found that these polymorphisms in miRNA were associated with the risk of GC; in addition, genotypes rs2292832 and rs3746444 were associated with survival rates of GC patients. Xiong XD [21] found that rs895819 in pre-miR-27a could alter the expression level of the miRNA and thus was correlated with the incidence of cervical cancer. A previous study showed correlations between genetic variants in miRNA and gastric lesions or even GC. One study investigated rs112310158 in hsa-miR-449a in Chinese population and revealed that GG genotype of rs112310158 had a higher risk of GC than other genotypes [22]. Jin X [23] analyzed genotypes of SNPs in mir-421 and found it to be significantly associated with GC susceptibility, lymphatic metastasis, and prognosis.

The expression level and regulatory function of miRNA depend on the orderly division of function of genes in miRNA biogenesis pathways. Proteins such as GEMIN4, AGO1, DROSHA, DICER, and their complex regulating miRNA biogenesis pathways are key components of miRNA maturation, transfer, and function. Proper cooperation of these proteins enables the expression of genes that regulate miRNA. Genetic variants in miRNA machinery genes could affect the maturation and regulatory function of miRNA by influencing the transcription ability of genes (UTR region) or protein function (exon region), thus manifesting as a change in tumor susceptibility and malignant behavior [8, 24]. Recent studies have already revealed a relationship between SNPs in miRNA machinery genes and several tumors including GC [13, 25–27], and investigation of variants in miRNA machinery genes could clarify the mechanism of the occurrence and development of GC and provide new basis for its clinical diagnosis and management. Our group speculates that genetic polymorphisms of the important miRNA machinery genes (*DICER*, *GEMIN4*, *DROSHA* and *AGO1*) could play a role in GC susceptibility and malignant behavior by affecting the maturity and functioning of miRNA.

This study analyzed the genotype and allele frequencies of four SNPs in miRNA machinery genes (*GEMIN4*, *DROSHA*, *DICER* and *AGO1*) in GC patients and healthy controls in Chinese Han population to investigate whether the genetic polymorphisms in these genes can affect the susceptibility, invasion and metastasis of GC. We found that among the chosen SNPs, the distribution of genotype and allele frequencies of rs3742330 in *DICER* and rs7813 in *GEMIN4* were significantly different between GC patients and healthy controls, indicating that genetic variants in *DICER* and *GEMIN4* were correlated with GC susceptibility in Chinese Han population. Tchernitsa O [28] analyzed the expression of *DICER* in adjacent normal and tumor samples of patients with GC by using immunohistochemistry and detected an elevated DICER level in GC tissues. However, another study using the same sample type and analytical method demonstrated a down regulation of DICER in GC tissues in both mRNA and protein levels [29]. There is no study demonstrating a definite association between *GEMIN4* and GC. Xie Y [26] investigated SNPs in miRNA machinery genes including *GEMIN4* (rs2740348) but found no significant correlation between this SNP in *GEMIN4* and GC pathogenesis. Despite the controversial results reported, it is clear that *DICER* and *GEMIN4* participated in the pathogenesis of tumors including GC, and polymorphisms in these genes could affect tumor susceptibility. Thus far, the influence of SNPs that we investigated in *DICER* and *GEMIN4* on GC susceptibility had rarely been reported. Rs3742330 in *DICER* had been reported to be associated with the risk of larynx cancer in Polish population [30]. Another study in Korean population showed a significantly increased risk of colon cancer in individuals with AG genotype of rs3742330 [31]. The location of rs3742330 in the 3’-UTR region of *DICER* may potentially influence the stability and expression of *DICER* through changing the binding capacity of regulatory miRNAs [32, 33]. But the mechanism underlying how rs3742330 modified GC susceptibility remains unclear. Our group conducted the *in-silico* analysis and found that rs3742330 was located in the hsa-miR-632 potential target sequence in *DICER* 3'UTR region, which might probably upregulate the expression of DICER. Rs7813 in *GEMIN4* was reported to be evidently associated with the risk of lung cancer [34], but another study showed no significant association of rs7813 with the risk of esophageal squamous cell carcinoma [35]. Our predicted analyses showed that rs7813 could alter the structure of GEMIN4 protein by turning Arginine into Cysteine and the alteration might reduce GEMIN4 expression. It was reported that rs7813 in *GEMIN4* could induce Arg to Cys substitution at the 1033 amino acid position through the C to T transition [34], which could then affect the function of miRNAs. Our study found a correlation between the two polymorphisms in *DICER* and *GEMIN4* and GC susceptibility, suggesting a predictive role of these SNPs in gastric carcinogenesis.

Furthermore we established a database of all the GC patients, including enormous clinical information such as tumor size, tumor stage, degree of differentiation, lymphatic metastasis and so on. Stratified analysis with all the clinical features revealed a notable correlation between rs3742330 (*DICER*) and rs7813 (*GEMIN4*) and the stage of GC, providing molecular markers of prognosis at an early stage. In addition, the TT genotype and the T allele of rs7813 (*GEMIN4*), and the AA genotype and A allele of rs636832 (*AGO1*) were related to lymphatic metastasis of GC. These three SNPs could be potential biomarkers for predicting the invasion and metastasis of GC. Previously, several researchers have reported the dysregulation and potential role of *DICER*, *GEMIN4* and *AGO1* in tumor progression, including GC. Down regulation of *DICER* has been reported to be highly correlated with tumor differentiation and lymph node invasion in GC tissues, while decrease of DICER was more common in GC cases with low tumor differentiation and lymph node metastasis [29]. Shi Z [36] further demonstrated the mechanism that DICER could process pre-miR-21 to mature miR-21, while the inhibitor of DICER (AC1MMYR2) blocked its ability for miRNA maturation and further suppressed proliferation, survival, and invasion in glioblastoma, breast cancer, and gastric cancer cells *in vivo*. According to an *in vitro* experiment, DEAD-box RNA helicase 6 (DDX6), which directly interacts with AGO1 in RNA-induced silencing complexes (RISC), was reported to down regulate miR-143/145 expression by prompting the degradation of its host gene product [37]. Thus far, no association has been found between GEMIN4 and GC progression. Consistent with our study, rs3742330 in *DICER* and rs7813 in *GEMIN4* were found to participate in tumor progression. Mi Na Kim [38] demonstrated that rs3742330 was associated with the survival of hepatocellular carcinoma patients, while another study reported that the G allele of rs3742330 was associated with lower aggressiveness of prostate cancer [39]. Yang PW [40] showed a borderline significant association between rs7813 in *GEMIN4* and the prognosis of esophageal squamous cell carcinoma (ESCC). In addition, *AGO1* is located at chromosome 1p35-p34 and frequently lost in human malignant tumors, and rs636832 is located in the intron of *AGO1*, which might influence the conformation and function of proteins or the splicing of precursor miRNA [41], but no study reported the effect of rs636832 in *AGO1* on tumor development, while current studies have not yet demonstrated a definite correlation between rs3742330 as well as rs7813 and GC invasion and metastasis. Our present study is the first to revealed an influence of the three SNPs in miRNA machinery genes on GC progression.

The results from this study demonstrated that genetic polymorphisms in miRNA machinery genes (*DICER*, *GEMIN4* and *AGO1*) affected the susceptibility and the invasion and metastasis of GC in Chinese Han population, extremely probably by affecting maturing and functioning of relevant miRNAs. We confirmed in a relatively large sample size that these polymorphisms participated in the development of GC and its malignant behavior, genetically proving the essential roles of these genes in tumorigenesis and progression of tumor. Follow-up studies with larger sample size are required to further verify the results and design innovative experiments and functional verification to investigate the specific mechanism by which polymorphisms in these miRNA machinery genes influence the maturation of miRNA and then participate in the genesis and development of GC. The subsequent research could further reveal the molecular mechanism of GC and provide new molecular markers for GC diagnosis and treatment.

## MATERIALS AND METHODS

### Study populations

The study involved 628 cases and 502 controls. The cases were from West China Hospital outpatient or inpatients with GC between July 2010 and July 2016. The diagnosis of GC was based on both clinical criteria and pathological confirmation. The controls included unrelated healthy individuals screened from the physical examination center of West China Hospital of Sichuan University. All the controls had no significant history of disease. The controls were matched with the cases in the age and gender and came from the same region and same period as the cases. All participants provided informed consent to participate in the study, and this study was approved by the ethical committee of West China Hospital of Sichuan University.

Genomic DNA was extracted from the peripheral blood of the participants by using QIAamp^®^ DNA Blood mini kit (Qiagen, Düsseldorf, Germany) following the manufacturer’s instructions. The samples were selected from patients with GC who had not been treated with chemotherapy but had been pathologically confirmed. Each sample used in the experiment had detailed clinical information and DNA met the requirements of concentration and purity.

### SNP selection and genotyping

Based on the data from the International HapMap Project (http://www.hapmap.org), dbSNP (http://www.ncbi.nlm.nih.gov/projects/SNP/), and miRBase registry (http://microrna.sanger.ac.uk), we identified 20 potential polymorphisms in the miRNA biogenesis pathway (Supplementary Table 1) that met the criteria of minor allele frequency (MAF) > 0.01 in Chinese population. Thirty subjects including 15 healthy individuals and 15 patients with GC were randomly involved in SNP screening by high resolution melting (HRM). Finally, only five GC-associated SNPs with a high frequency (>0.1) of the minor allele were selected (rs3742330 in *DICER*, rs3744741 in *GEMIN4*, rs7813 in *GEMIN4*, rs10719 in *DROSHA*, and rs636832 in *AGO1*).

The isolated DNA was stored in a freezer at -80°C. Genotyping of the SNPs was performed by the HRM method. The data were analyzed using the LightCycler^®^480 Gene Scanning software (v1.2, Roche Diagnostics, Bavaria, Germany). Polymerase chain reaction (PCR) amplifications were conducted in the LightCycler^®^ 480 (Roche Diagnostics). The PCR reaction mixture (20 μL) included the following: 0.5 μL forward primer (10 μM), 0.5 μL reverse primer (10 μM), 0.2 μL Hot Star Taq^®^ Plus DNA Polymerase (5 U/μL), 1 μL 20×EVA-GREEN, 2 μL dNTP (10 mM), 1 μL genomic DNA (10 ng/μL), 2 μL MgCl_2_ (25 mM), 2 μL 10×buffer, and 10.8 μL H_2_O. Real-time PCR was performed with the following conditions: an initial denaturation at 95°C for 15 min, followed by 50 cycles of denaturation at 95°C for 10 s, annealing at 60°C for 15 s, extension at 72°C for 25 s. Following the completion of the cycle program, PCR products were denatured at 95°C for 1 min and cooled to 40°C for 1 min to form double-stranded DNA. The HRM analysis was then performed by gradually increasing the temperature from 65°C to 95°C at a rate of 0.01°C/s. Three DNA samples with known genotypes were run simultaneously in each experiment as a reference, and 10% of the samples were randomly selected to genotype twice; all results were identical.

### DNA sequencing

PCR products were purified using shrimp alkaline phosphatase (SAP). Sequencing primers for the five SNPs were the same as primers in PCR. Nucleotide sequencing was detected by BigDye Terminator v3.1 Cycle Sequencing Kit and ABI 3130 genetic analyzer (Applied Biosystems, California, USA).

### *In-silico* analysis of microRNA-binding and function prediction

The mature human microRNA sequences were obtained from the microRNA database (miRBase) (http://microrna.sanger.ac.uk). A region comprising the rs3742330 plus 15 bp 5' and 3' was included for analyzing hybridization of putative microRNAs using miRanda software with default parameters. The predicted analysis for rs7813 and rs636832 was conducted using Polyphen2 online software (http://genetics.bwh.harvard.edu/pph2/).

### Statistical analysis

The Goodness-of-fit chi-square test (χ^2^) was used for testing Hardy-Weinberg Equilibrium (HWE) with cases and controls. Differences in demographic characteristics were assessed by Student’s t-test (for continuous variables) or χ^2^ test (for categorical variables). Logistic regression was used to analyze the associations between SNPs and susceptibility of GC, adjusted by sex, age, smoking status, and drinking status. All the statistical analyses were two-sided and *P* < 0.05 was set as a criterion for statistical significance. All statistical analyses were performed using SPSS statistical software (version 20.0, SPSS Inc., USA).

## SUPPLEMENTARY MATERIALS FIGURES AND TABLE


