# Supplementary material for: Association between SNPs in microRNA machinery genes and gastric cancer susceptibility, invasion, and metastasis in Chinese Han population
Source: Oncotarget. 2017 Sep 23;8(49):86435–46. doi: 10.18632/oncotarget.21199 (PMC5689696; doi:10.18632/oncotarget.21199)
Supplement: Supplementary file 1 [file oncotarget-08-86435-s001.pdf]

Association between SNPs in microRNA machinery genes and gastric cancer susceptibility, invasion, and metastasis in Chinese Han population

SUPPLEMENTARY MATERIALS

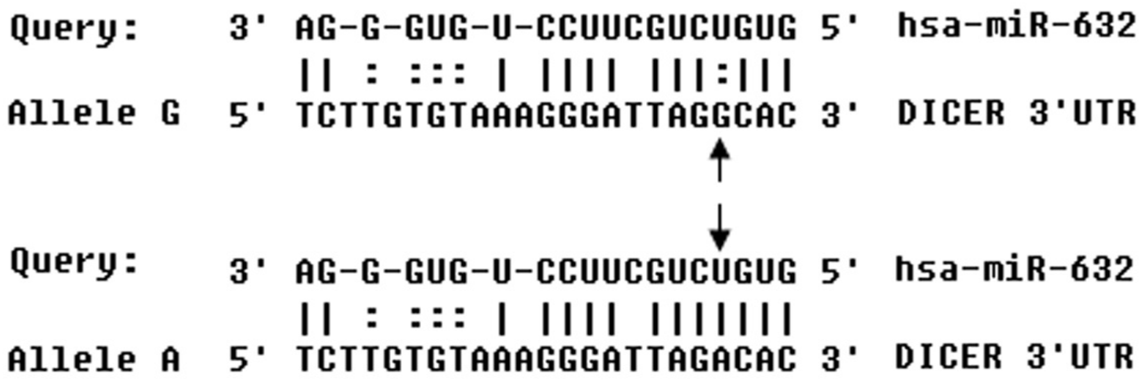

Supplementary Figure 1: *In-silico* analysis of microRNA-binding. rs3742330 was located at the fourth nucleotides from 5' end of the has-miR-632. The arrows indicated the position of rs3742330.

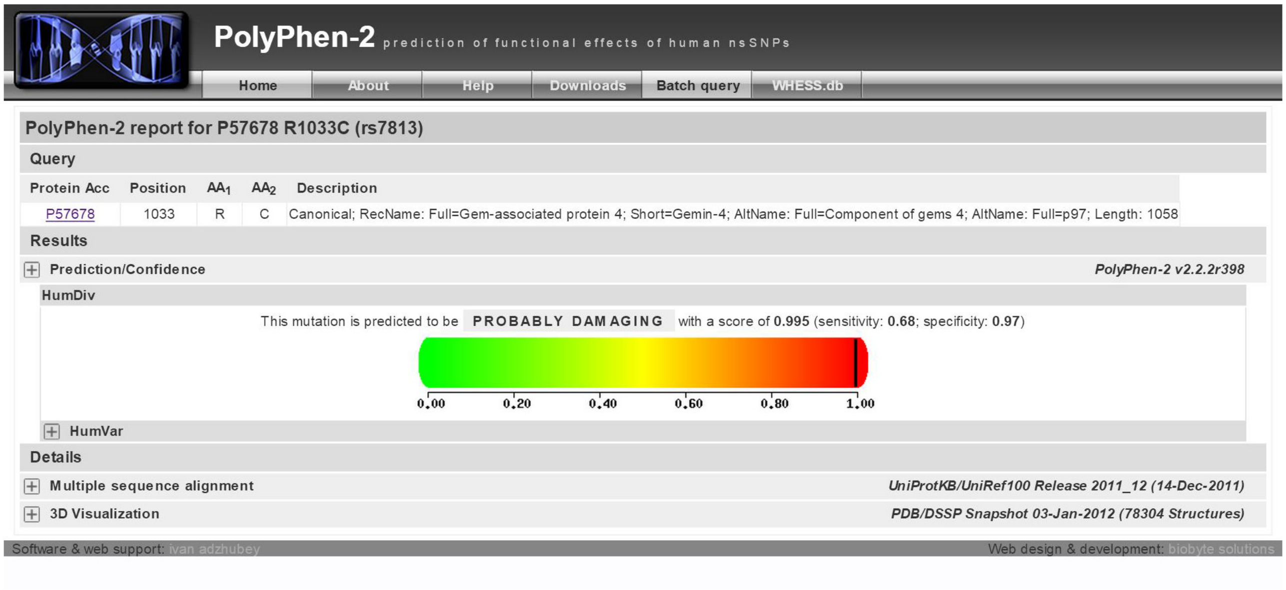

Supplementary Figure 2: The predicted analysis for rs7813.

Supplementary Table 1: MiRNA-related genes and polymorphisms evaluated in this study

Gene	SNP ID	Position	Major/minor allele	MAF%
DROSHA	rs642321	3'UTR	C/T	0.467
	rs6877568	5'UTR	A/C	0.216
	**rs10719**	**3'UTR**	**C/T**	**0.233**
DGCR8	rs3757	3'UTR	A/G	0.178
	rs720012	3'UTR	A/G	0.444
	rs417309	3'UTR	A/G	0.100
	rs1640299	3'UTR	G/T	0.222
RAN	rs14035	3'UTR	C/T	0.178
DICER	**rs3742330**	**3'UTR**	**A/G**	**0.267**
AGO1	rs595961	intron	A/G	0.128
	**rs636832**	**intron**	**A/G**	**0.198**
AGO2	rs4961280	promotor	A/C	0.111
GEMIN4	rs104591	3'UTR	A/G	0.148
	rs104581	Coding exon	C/T	0.133
	**rs7813**	**Coding exon**	**C/T**	**0.289**
	rs2740349	Coding exon	A/G	0.211
	**rs3744741**	**Coding exon**	**C/T**	**0.267**
GEMIN3	rs197412	Coding exon	C/T	0.333
HIWI	rs1106042	Coding exon	A/G	0.122
	rs10773771	3'UTR	G/T	0.409
